# Anticancer Effects of Propionic Acid Inducing Cell Death in Cervical Cancer Cells

**DOI:** 10.3390/molecules26164951

**Published:** 2021-08-16

**Authors:** Chau Ha Pham, Joo-Eun Lee, Jinha Yu, Sung Hoon Lee, Kyung-Rok Yu, Jaewoo Hong, Namki Cho, Seil Kim, Dukjin Kang, Soojin Lee, Hee Min Yoo

**Affiliations:** 1 Biometrology Group, Korea Research Institute of Standards and Science, Daejeon 34113, Korea; pham.ha.chau@hotmail.com (C.H.P.); stapler@kriss.re.kr (S.K.); djkang@kriss.re.kr (D.K.); 2 Department of Microbiology and Molecular Biology, Chungnam National University, Daejeon 34134, Korea; leesoojin@cnu.ac.kr; 3 Department of Physiology, Daegu Catholic University School of Medicine, Daegu 42472, Korea; jsmlo@hanmail.net (J.-E.L.); jhong@cu.ac.kr (J.H.); 4 Chemical Kinomics Research Center, Korea Institute of Science and Technology, Seoul 02792, Korea; yuj@kist.re.kr; 5 College of Pharmacy, Chung-Ang University, Seoul 06974, Korea; sunghoonlee@cau.ac.kr; 6 Department of Agricultural Biotechnology, Research Institute of Agriculture and Life Sciences, Seoul National University, Seoul 08826, Korea; cellyu@snu.ac.kr; 7 Research Institute of Pharmaceutical Sciences, College of Pharmacy, Chonnam National University, Gwangju 61186, Korea; cnamki@chonnam.ac.kr; 8 Convergent Research Center for Emerging Virus Infection, Korea Research Institute of Chemical Technology, Daejeon 34114, Korea; 9 Department of Bio-Analytical Science, University of Science & Technology (UST), Daejeon 34113, Korea

**Keywords:** cervical cancer, short-chain fatty acids, propionic acid, reactive oxygen species, HeLa

## Abstract

Recent studies found that short-chain fatty acids (SCFAs), which are produced through bacterial fermentation in the gastrointestinal tract, have oncoprotective effects against cervical cancer. The most common SCFAs that are well known include acetic acid, butyric acid, and propionic acid, among which propionic acid (PA) has been reported to induce apoptosis in HeLa cells. However, the mechanism in which SCFAs suppress HeLa cell viability remain poorly understood. Our study aims to provide a more detailed look into the mechanism of PA in HeLa cells. Flow cytometry analysis revealed that PA induces reactive oxygen species (ROS), leading to the dysfunction of the mitochondrial membrane. Moreover, PA inhibits NF-κB and AKT/mTOR signaling pathways and induces LC3B protein levels, resulting in autophagy. PA also increased the sub-G1 cell population that is characteristic of cell death. Therefore, the results of this study propose that PA inhibits HeLa cell viability through a mechanism mediated by the induction of autophagy. The study also suggests a new approach for cervical cancer therapeutics.

## 1. Introduction

Among the various types of cancers known to mankind, cervical cancer is the third most common form of cancer found in cancer patients and the fourth leading cause of death among women worldwide [1,2]. The link between sexual activity and the disease was confirmed when human papillomavirus (HPV) was identified as the major cause of cervical cancer [3]. Among all cervical cancer cases, 70% are caused by two types of HPV, 16 and 18, whereas the remaining 30% are caused by other high-risk HPV types [3]. HPV oncogenes E6 and E7 are required for the proliferation of cervical carcinomas and cervical carcinoma cell lines [4]. HPV infections in the cervix causes host genome alterations, promotes the silencing of tumor-suppressor factors, and induces abnormal tumor-promoting factors [5].

Probiotics are known for their ability to benefit the host’s health [6,7]. Recently, significant evidence has been reported regarding how probiotic bacteria and commensal bacterial flora that form colonies in the body influence human health or pathological conditions such as cancer [8]. Gut microbiota, especially probiotic bacteria, have been reported as central pieces in the promotion of anticancer effects and immunomodulation [9,10]. Probiotic bacteria, such as *Bifidobacterium* or *Lactobacillus*, commonly exist in human breast milk or fermented milk and have been reported to be linked to numerous health benefits [11,12]. It has also been indicated that vaginal microbiome alterations serve a pivotal role in the occurrence and development of cervical cancer [13]. Furthermore, recent reports have described the significant roles of abnormal vaginal microbiota in cervical cancer development [14,15]. In general, cervicovaginal microbiota are host to an abundance of the *Lactobacillus* genus [16,17]. Numerous studies have investigated the anticancer effects of lactic acid bacteria (LAB). In particular, multiple reports have proposed several mechanisms regarding the anticancer activities of LAB, including the production of anticancer metabolites (such as lactic acid and short-chain fatty acids) as well as the inhibition of cancer cells [18].

Several papers studied short-chain fatty acids (SCFAs), metabolites produced in the gut microbiota that helps sustain human homeostasis by contributing to gut integrity, energy metabolism, hormone production, and immune functions [13,19,20]. However, few studies have investigated the antimicrobial and immune modulatory activities of short-chain fatty acids (SCFAs) and lactic acid produced by vaginal microbiota, which highlights the potential of the two substances as biomarkers of disease [19,21]. Recent studies have reported the oncoprotective effects of free fatty acid receptor 2 (FFAR2), the ligands of which are SCFAs, on several types of neoplasia [22]. Nevertheless, it is still unknown whether SCFAs, including propionic acid, have anticancer effects on uterine cervical neoplasm, and the mechanisms through which such effects take action are also unknown [22]. 

Autophagy is a necessary part of cellular processes in multicellular organisms [23]. The initiation of autophagy removes cytoplasmic components to the lysosome [24]. Furthermore, as autophagy reduces the number of damaged proteins and organelles in cells, it contributes to cellular homeostasis. Additionally, dysfunctions regarding autophagy can arise from various disorders, such as neurodegenerative diseases, metabolic syndrome, and tumorigenesis [25]. Although autophagy can help in ensuring cell survival, it can also lead to cell death through certain conditions. This is because cell death is also associated with autophagy and nonlysosomal vesiculate cell death, in addition to apoptosis [26]. Many researchers have published papers reporting how several cancer cells exhibit the activation of autophagy or autophagic cell death in response to various types of anticancer treatment [27]. For example, in breast cancer cells, it was reported that autophagic cell death was induced by tamoxifen [28]. The natural product, soybean B-group triterpenoid saponins, were also reported to stimulate autophagy in colon cancer cells [29]. Moreover, PA also induced autophagic cell death by increasing both LC3 puncta formation and the conversion of LC3-I to LC3-II in colon cancer cells [30]. Therefore, induced autophagic pathways in cancer cells have been important factors in the development of new potential drug targets [31]. Moreover, both apoptosis and autophagy are closely associated with, and regulated by, the same types of cell stress, such as reactive oxygen species (ROS) [32,33]. In this study, we discovered the therapeutic potential of PA against cervical cancer cell lines. 

## 2. Results and Discussion

### 2.1. Cytotoxic Effect of PA in HeLa Cells

**Figure 1:**
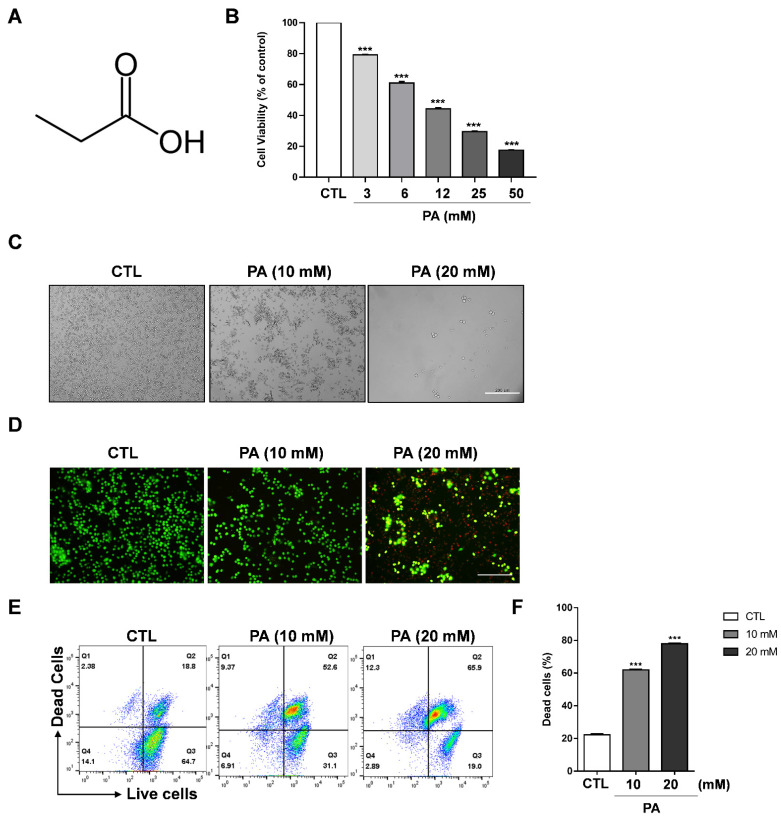
PA-induced cytotoxic effects on HeLa cells. (**A**) Structure of propionic acid. (**B**) Cell viability as measured through MTS assays. HeLa cells were exposed to various concentrations of PA. (**C**) Cell morphologies taken after 48 h of treatment with PA (scale bar: 200 μm). (**D**) Live and dead assay performed following PA treatment of HeLa cells. The cells were stained with calcein AM and ethidium homodimer-1 and images were taken using a fluorescent microscope (scale bar: 200 μm). (**E**) Assessment of the live and dead assay using a flow cytometer. (**F**) Quantification of dead cells. Values indicate means ± SEM. (*n* = 3, *** *p* ≤ 0.001).

To investigate the viability of propionic acid (PA) to regulate the growth of cervical cancer cell lines (such as HeLa, CaSki, and SiHa cells) and the normal cell line BEAS-2B, these cells were treated with PA at concentrations ranging from 0 mM to 50 mM in 96-well plates for 48 h. CaSki and SiHa exhibited dramatically reduced cell viability in contrast to BEAS-2B (Supplementary Materials Figure S1). The structure of PA is shown in Figure 1A. A cell viability assay was performed to evaluate cell cytotoxicity, which showed that cell viability was significantly inhibited to 42.7% at a PA concentration of 12 mM, and to 31.5% at 25 mM in HeLa cells (Figure 1B). Moreover, CaSki and SiHa exhibited dramatically reduced cell viability in contrast to BEAS-2B, which is a nontumorigenic lung epithelial cell line (Supplementary Materials Figure S1). As a result, we used 10 mM and 20 mM PA concentrations for microscopic analysis and further experiments. For the microscopic analysis, HeLa cells were treated with 10 mM and 20 mM PA, which gradually changed the morphology of the cell in a concentration-dependent manner (Figure 1C). It is noteworthy that the cells were round-shaped in 20 mM after 48 h, which is a typical feature of apoptosis. Moreover, a live cell and dead cell assay was performed with a mixture of two fluorescent dyes: calcein, a green dye for live cells, and ethidium homodimer-1 (EthD-I), a red dye for dead-cells. After washing and staining with calcein and EthD-1, the cells were analyzed via imaging fluorescence microscopy and flow cytometry. Figure 1D indicates that PA dramatically decreased the number of live cells (green color) and increases the number of dead cells (red color) when treated with 20 mM PA. This was further supported by results obtained using a flow cytometer: the amount of dead cells increased by approximately 52.6% in 10 mM PA and 65.9% in 20 mM PA compared to the control group (Figure 1E,F). Therefore, our data suggests that PA dramatically suppresses the viability of cervical cancer cell lines.

### 2.2. PA-Induced Apoptosis in HeLa Cells

**Figure 2:**
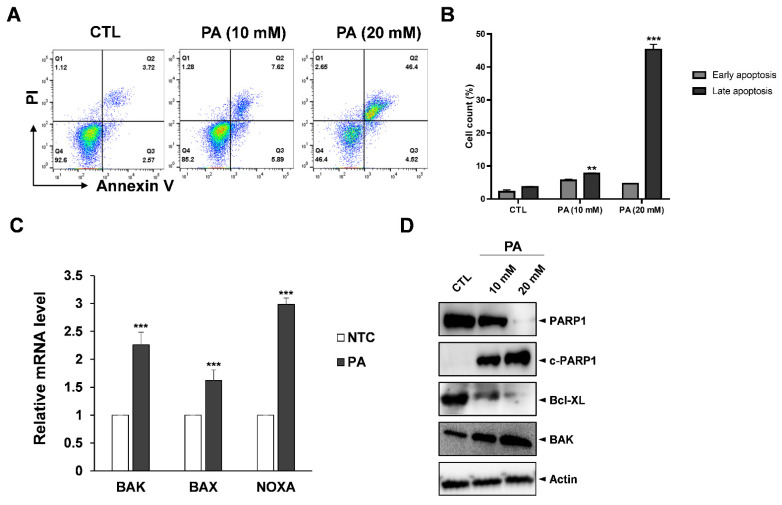
PA-induced apoptosis in HeLa cells. (**A**) Apoptosis population after treating HeLa cells with PA for 48 h. The cells were harvested, stained with PI and Annexin V, and then measured by flow cytometry. (**B**) Quantification of the early and late apoptosis populations. (**C**) RT-PCR results with apoptotic markers such as BAK, BAX and NOXA and normalized by actin. (**D**) Protein expression of cleaved PARP1, PARP1, BAK, and BCL-XL as examined by Western blot analysis. Values indicate means ± SEM. (*n* = 3, ** *p* ≤ 0.01, *** *p* ≤ 0.001).

As suppression of cell growth is related to apoptosis, we performed fluorescence-activated cell sorting (FACS) analysis with Annexin V/PI double straining to examine whether PA affects apoptosis. Annexin V is a member of the annexin family that binds to phosphatidylserine (PS) when PS translocates from the intermembrane to the outer membrane during early apoptosis, a process of which is dependent on calcium. Propidium iodide (PI) can bind to damaged DNA to assist in distinguishing between necrotic and apoptotic cells during late apoptosis stages [34]. To prove our apoptosis assay system works, we tested apoptosis analysis with positive controls, such as paclitaxel and etoposide, using HeLa cells [35,36]. Two anticancer agents, paclitaxel and etoposide, treated for 48 h, which was the same condition with PA, dramatically induced apoptosis in HeLa cells (Supplementary Materials Figure S2). Then, HeLa cells exposed with 20 mM of PA had dramatic increases in late apoptosis to 46.4% compared to the control sample (Figure 2A,B). Next, the anticancer effects of PA were evaluated at the molecular level. Primers were designed to amplify various apoptosis-related genes such as BAK, BAX and NOXA, which are well-known proapoptosis regulators. RT-PCR data showed that all three related apoptosis genes increased in amount; NOXA in particular increased more than threefold compared to the control samples. On the other hand, BAK and BAX increased by 2.26 and 1.6 times, respectively (Figure 2C). Moreover, Bcl-XL, which is a prosurvival protein, had significantly reduced expression, whereas cleaved-PARP and BAK increased due to PA treatment in a dose-dependent manner (Figure 2D).

The Bcl-2 protein family are highly important in the regulation of the mitochondrial pathway of apoptosis. The family includes three subclasses: the proapoptotic BH3-only proteins (Bad, Noxa), the prosurvival Bcl-2-like proteins (Bcl-2, Bcl-XL), and proapoptotic proteins (BAK, BAX) [37]. As their names suggest, prosurvival Bcl-2/Bcl-XL possess the ability to bind and sequester to pro-apoptotic proteins. However, it has been proposed that during apoptosis BH3-only proteins act as activators of BAK and BAX. Thus, BAK and BAX become active, resulting in the outer mitochondrial membrane being punctured and ultimately leading to apoptotic cell death [38]. According to our data, PA blocks pro-survival Bcl-XL proteins downstream and upregulates NOXA to activate BAK and BAX, resulting in the HeLa cells undergoing apoptosis.

### 2.3. Intracellular ROS Generation in HeLa Cells Triggered by PA

**Figure 3:**
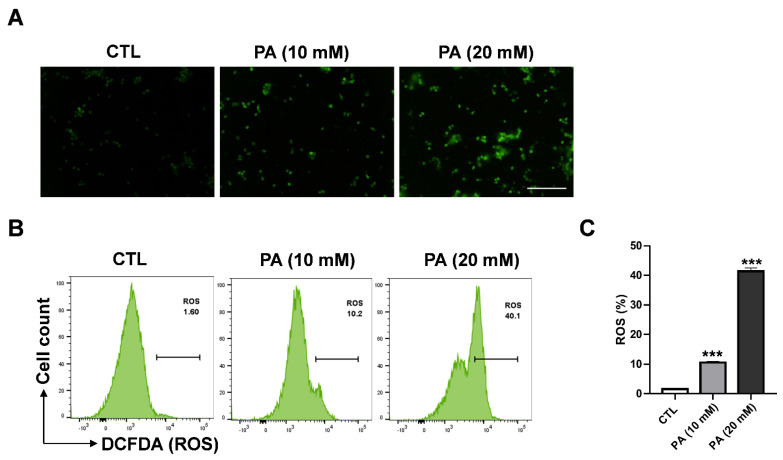
PA-induced intracellular ROS generation in HeLa cells. (**A**) PA-induced ROS as observed via fluorescence microscopy (scale bar: 200 μm). (**B**) Effects of 10 mM and 20 mM of PA for 48 h on ROS levels as measured using DCF-DA and a flow cytometer. (**C**) Quantification of ROS levels. Values indicate means ± SEM. (*n* = 3, *** *p* ≤ 0.001).

Reactive oxygen species (ROS) regulate various important progression stages in biological responses that serve to maintain the redox balance of activate cellular cell signaling or transcription pathways [39]. Nevertheless, high levels of ROS accumulation can lead to cell damage and activate cell death signal pathways as apoptosis. To determine whether PA-induced apoptosis is dependent on ROS, we conducted fluorescence spectroscopy to examine how ROS generation in HeLa cells is affected by PA treatment. For the experiment, DCFH-DA was used as a fluorescent probe. Fluorescence microscopy was applied to measure the accumulation of ROS between treated and nontreated groups. As a result, it was clearly shown that the enhancement of ROS level was dependent on PA concentration (Figure 3A) In addition, flow cytometry analysis showed that HeLa cells treated with PA of 10 mM and 20 mM concentrations significantly engendered ROS at 10.2% and 40.2%, respectively, compared to 1.6% in the control sample, as shown in Figure 3B,C.

### 2.4. PA-Induced Mitochondrial Membrane Dysfunction

**Figure 4:**
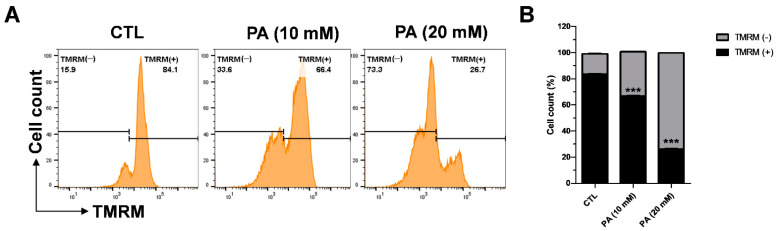
PA-induced intracellular ROS generation in HeLa cells. (**A**) Effects of 10 mM and 20 mM PA for 48 h on mitochondrial membrane potential measured using a TMRM reagent and a flow cytometer. (**B**) Quantification of mitochondrial membrane potential. Values indicate means ± SEM. (*n* = 3, *** *p* ≤ 0.001).

The enhancement of ROS causes oxidative stress, which triggers mitochondrial membrane dysfunction and induces apoptosis. Therefore, we examined the effects of PA on the mitochondrial membrane potential by measuring tetramethylrhodamine methyl ester (TMRM). A cervical cancer cell line was exposed to 10 mM and 20 mM PA for 48 h following probing with 100 nM TMRM for 30 min at 37 °C. Afterwards, the intensity of TMRM binding in the healthy membrane was calculated via flow cytometry. As displayed in Figure 4A,B, TMRM (+) indicates the intensity of TMRM, which dramatically dropped to 66.4% at 10 mM and 26% at 20 mM in contrast to the control value of 84%. These results demonstrate that the depolarization of the HeLa mitochondrial membrane that leads to dysfunction is related to PA. 

### 2.5. Inhibition Analysis of NF-κB

There are five transcription factors in the Nuclear factor -kappa B (NF-kB) protein family (p65/RelA, RelB, c-Rel, NF-kB1/p50, and NF-kB/p52), which are involved in regulating hundreds of genes in cell growth, differentiation, and apoptosis processes [40,41]. Under basal conditions, NF-KB complexes are inactive in the cytoplasm and bind to inhibitor proteins that inhibit NF-kB (IkB) proteins, most notably IkBa [42]. In response to stimuli signals, phosphorylated IkBa is degraded by ubiquitination, which is controlled by the upstream kinase (IKK) [42,43]. Subsequently, NF-kB p65/p50 dimers are translocated into the nucleus, binding to DNA and inducing inflammatory and antiapoptotic genes [44,45].

**Figure 5:**
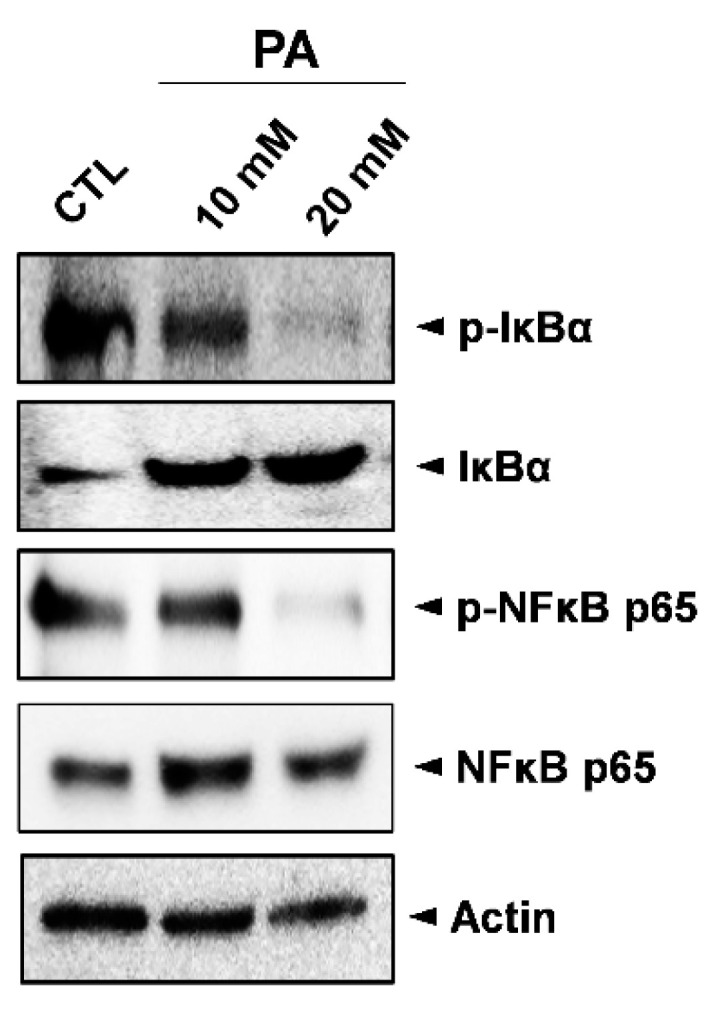
Inhibition of the NF-κB signaling pathway in HeLa cells by PA. Whole cell lysates were used to determine the expression levels of IκBα, p-IκBα (Ser32), NFκB p65, and p-NFκB p65 (Ser536) after treating cells with 10 mM and 20 mM PA for 48 h.

In order to investigate the effects of PA modulation on the NF-kB signaling pathway, Western blot analysis was conducted to examine expressions of phosphorylated and unphosphorylated IκBα (p-IκBα, IκBα), as well as phosphorylated and unphosphorylated NF-κB p65 (p-NFκB p65/NFκB p65). As shown in Figure 5, the expressions of both p-IκBα and p-NFκB p65 were markedly decreased following treatment with PA when compared to the control sample. This suggests that the inhibition of the NF-κB signaling pathway is involved in PA-induced cell death in HeLa cells.

### 2.6. Activation of Autophagy by PA

**Figure 6:**
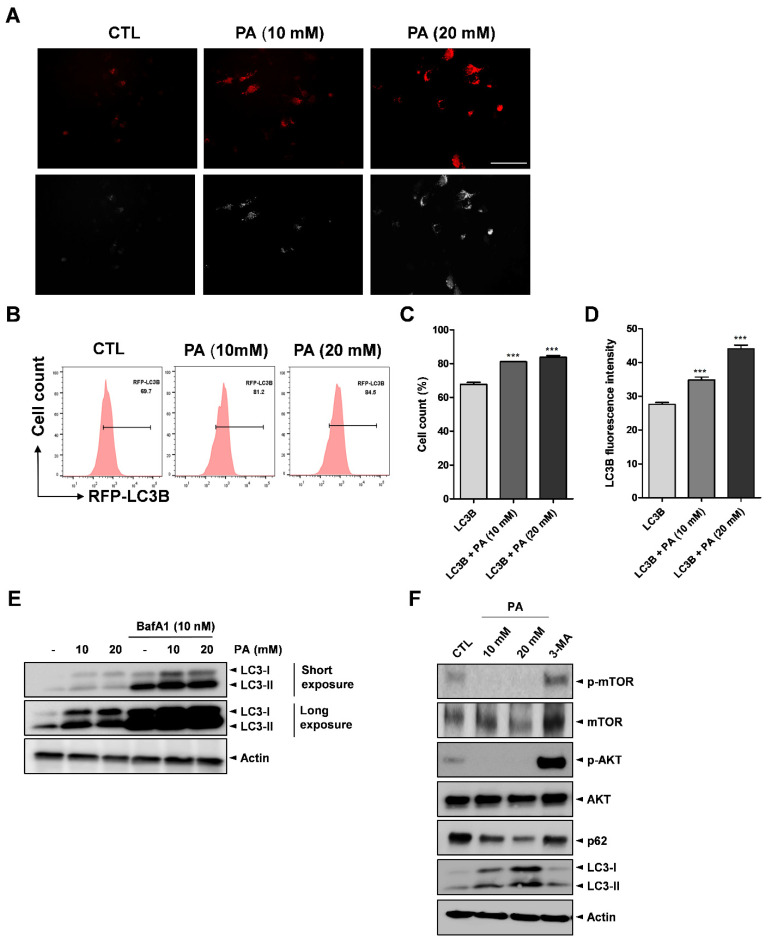
PA-activated autophagy. RFP-LC3B was transfected into HeLa cell for 16 h then treated with PA for 24 h. (**A**) Fluorescence microscopy results used to analyze the effects of PA with LC3B puncta (white arrows) formation (scale bar: 200 μm). (**B**) Effects of 10 mM and 20 mM PA on LC3B puncta formation using RFP-LC3B and RFP as measured using a flow cytometer. (**C**) Quantification of RFP-LC3B. (**D**) Quantitative analysis of LC3B median fluorescence intensity. (**E**) Representative images of immunoblot analysis results of LC3B obtained from autophagy flux assays. The cells were pretreated with 10 nM Bafilomycin A1 (BafA1) for 4 h then treated with PA for 24 h. (**F**) Expressions of p-mTOR (Ser2448), mTOR, p-AKT (Ser473), AKT, p62, LC3B, and actin as assessed by Western blot analysis. 5 mM of 3-Methyladenine (3-MA) were pretreated for 4 h then treated with PA for 24 h. *** *p* < 0.001.

Several studies revealed that SCFA treatment on cancer cells triggers autophagy activation [46,47]. Moreover, it has been reported that the involvement of autophagy in key cellular processes, such as metabolic reprogramming, metastasis, treatment resistance, and cell death, can lead to the development of tumors [48]. Therefore, we tested whether PA could activate autophagy in cervical cancer cells. RFP-labeled microtubule-associated protein-1 light chain 3 (LC3) was transfected into HeLa cells treated with different PA concentrations. After 24 h, fluorescence microscopy and flow cytometry analysis were conducted to verify and examine the cells. As shown in Figure 6A, PA stimulated puncta accumulation (white arrows), which implicates LC3-II, in the 10 mM and 20 mM samples compared to the control. Additionally, the flow cytometry analysis results showed that PA increased the percentage of RFP-LC3B from approximately 69.7% to 84.5%, as shown in Figure 6B,C, and the median fluorescence intensity of LC3B was also increased (Figure 6D). The most popular assay for assessing autophagy flux involves monitoring endogenous LC3-I or LC3-II by Western blotting [49,50,51,52]. The autophagy flux assay revealed that PA increased LC3 levels and autophagic flux. Moreover, cotreatment of PA with BafA1 (an inhibitor of fusion between autophagosomes and lysosomes) caused further accumulation of LC3-II (Figure 6E). Moreover, we performed Western blotting to detect the expression of p62 and LC3B proteins as mediated by PA in the HeLa cells. As a result, it was found that LC3-II was upregulated, whereas p62 was downregulated, as shown in Figure 6F.

Next, we explored the effects of PA-activated autophagy in inhibiting autophagy. 3-methyladenine (3-MA), a popular autophagy inhibitor drug that is widely used in the study of autophagy and its roles, was utilized in this study to inhibit autophagy in the HeLa cells. The mechanism of 3-MA in autophagy is well known in that 3-MA suppresses autophagosome formation through the inhibition of type III phosphatidylinisitoi 3-kinases (PI-3K). Thus, we treated 5 mM of 3-MA to HeLa cells for 4 h, which was followed by exposure to 20 mM PA. Western blot analysis was conducted to evaluate the effects of PA on protein expression. According to the data in Figure 6F, PA increased the expression of the LC3B protein in the PA treatment group compared to the untreated group. Notably, PA induced more LC3B proteins with 3-MA treatment compared to the group that did not include the 3-MA treatment stage. 

mTOR is a kinase that serves as a downstream target of the AKT (AKR mouse thymoma kinase) pathway, which modulates autophagy [53]. To determine the role of AKT/mTOR signaling in PA-induced autophagy, we examined the expression levels of phosphorylated AKT and mTOR via Western blotting after the HeLa cells were treated with PA. The Western blotting results showed that both p-AKT and p-mTOR were reduced after PA treatment, inhibiting the AKT/mTOR pathway, whereas treatment with 3-MA reversed the inhibitory effect (Figure 6F). Previously, HeLa cells exposed to 10 mM and 20 mM PA showed dramatic increases compared to the control sample (Figure 3A,B). When cotreated with PA and 3-MA (an autophagy inhibitor), PA-induced cell death was compromised (Supplementary Materials Figure S3). This explains the mechanism of apoptosis via the induction of autophagy. Consequently, our data strongly suggest that PA activates autophagy by blocking the AKT and mTOR signaling pathway in cervical cancer cells.

### 2.7. PA-Induced Cell Cycle Distribution in HeLa Cells

**Figure 7:**
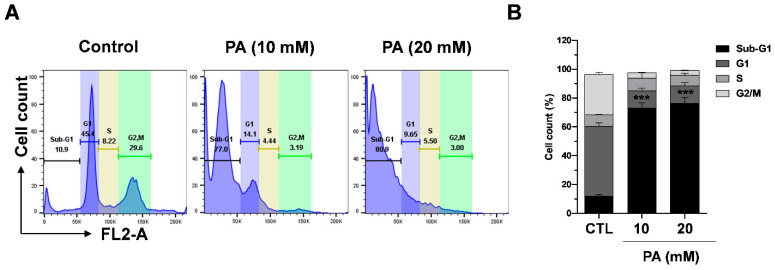
PA-induced cell cycle distribution in HeLa cells. (**A**) Percentage of cells in the G1, S, and G2/M phases after treatment with PA. The cells were analyzed using a flow cytometer. (**B**) Analysis of the cell cycles of the cells from the three experiments. *** *p* < 0.001 versus the control group.

The intracellular signaling pathway known as the PI3K/AKT/mTOR pathway is highly important in controlling the cell cyle [54]. In the present study, we demonstrated that PA exhibits inhibitory functions on the mTOR and AKT pathway. Thus, we next evaluated whether the effects of PA were related to cell cycle regulation. To assess the effects of PA, HeLa cells were treated with PA dose-dependently, and the cell cycle was then analyzed by performing flow cytometry. The results show that treatment with 10 mM and 20 mM PA significantly increased the sub-G1 phase from 10.9% to 77.0% and 80.9%, respectively. The findings indicate that PA induced sub-G1 phase cell populations, which are apoptotic cells, in HeLa cells Figure 7A,B.

## 3. Materials and Methods

### 3.1. Cell Culture

The HeLa, CaSki, and SiHa cells used in this study were purchased from the Korean Cell Line Bank (KCLB, Seoul, South Korea). BEAS-2B cells was purchased from the American Type Culture Collection (ATCC) (Manassas, VA, USA). The cell culture medium (Dulbecco’s modified Eagle medium, DMEM, Thermo Fisher Scientific, Waltham, MA, USA) containing 10% fetal bovine serum (FBS, Thermo Fisher Scientific, Waltham, MA, USA) and 1% penicillin-streptomycin, Thermo Fisher Scientific, Waltham, MA, USA) was used in accordance with the information suggested by KCLB or ATCC. The cells were cultured in an incubator in 5% CO_2_ at 37 °C. Subcultures were generated using a trypsin-EDTA solution when the cell density reached 80–90%.

### 3.2. Cell Viability Assay 

HeLa cells were seeded in 96 well plates up to a density of 6 × 10^4^ cells in each well then exposed to PA of various concentrations after 24 h. A cell viability assay was performed after 48 h of PA treatment using the CellTiter 96 AQueous One Solution Cell Proliferation Assay Kit (Promega, Madison, WI, USA). The cells were incubated with solution reagents for 2 h at 37 °C and absorbance was measured at 490 nm using a Synergy HTX Multi-Mode microplate reader (BioTek Instruments, Inc., Winooski, VT, USA).

### 3.3. Microscopy

For microscopy analysis, HeLa cells were seeded in 6-well plates up to a density of 1 × 10^6^ cells in each well and subsequently treated with PA (10 and 20 mM). After 48 h, cell morphology was observed under a phase 400x magnification contrast microscope (Olympus, Tokyo, Japan), as described in a previous paper written by the authors of this study [55].

### 3.4. Mitochondrial Membrane Potential (Δψm) Assay 

To measure the mitochondrial membrane potential, the HeLa cells were seeded in 6-well plates up to a density of 1 × 10^6^ cells in each well and the cells were treated with PA (10 mM and 20 mM) for 48 h. Afterwards, the cells were harvested and washed twice with cold phosphate-buffered saline (PBS) (Corning, Manassas, VA, USA). The cells were incubated with 100 nM TMRM (Thermo Fisher Scientific, Waltham, MA, USA) at 37 °C for 30 min. After incubation, the cells were washed again, resuspended in 2% FBS in PBS buffer, and then measured and analyzed using a flow cytometer (BD FACSVerse, BD Biosciences, San Jose, CA, USA) and the FlowJo software (Version 10, TreeStar, Ashland, OR, USA).

### 3.5. Measurement of Reactive Oxygen Species (ROS)

To measure reactive oxygen species, the HeLa cells were seeded in 6-well plates up to a density of 1 × 10^6^ cells in each well and the cells were treated with PA (10 mM and 20 mM) for 48 h. Intracellular ROS levels were detected using 2′,7′-dichlorodihydrofluorescein diacetate acetyl ester (DCFDA) (Thermo Fisher Scientific, Waltham, MA, USA). The cells were incubated with DCFDA (1 μM) at room temperature for 30 min, washed with PBS, and resuspended in FACS buffer (PBS supplemented with 1% FBS). Intracellular fluorescence was then analyzed using a flow cytometer (BD FACSVerse, BD Biosciences, San Jose, CA, USA) and the FlowJo software (Version 10, TreeStar, Ashland, OR, USA).

### 3.6. Apoptosis Analysis 

To evaluate the apoptosis cell population, the HeLa cells were seeded in 6-well plates up to a density of 1 × 10^6^ cells in each well and the cells were treated with PA (10 mM and 20 mM) for 48 h. Then, we used the Annexin V APC/PI Apoptosis Detection Kit (Biolegend, San Diego, CA, USA). After treating HeLa cells with 10 mM and 20 mM PA for 48 h in 6-well plates, the cells were harvested, washed twice with cold BioLegend Cell Staining Buffer, then resuspended cells in Annexin V binding buffer. 5 μL of APC Annexin V and 10 μL of PI solution were added to the cells and the cells were incubated for 20 min at room temperature, in the dark.

### 3.7. Western Blot Analysis

HeLa cells were incubated in PA (10 mM and 20 mM) for 48 h in 6-well plates up to a density of 1 × 10^6^ cells in each well. The cells were subsequently washed with cold PBS then lysed with RIPA buffer containing 0.1 mg/mL phenylmethylsulfonyl fluoride (PMSP), NEM, and Protease Inhibitor Cocktail. Cell lysates were centrifuged at 13,000 rpm, 4 °C for 15 min. Equal amounts of protein were resolved by 10% SDS-PAGE then transferred to a polyvinylidene difluoride (PVDF) membrane (Millipore Corp., Boston, MA, USA). The proteins were subsequently probed with specific primary antibodies in 3% BSA containing triton at 4 °C. Specific primary antibodies of cleaved PARP1 (194C1439), BAK (AT38E2), BCL-XL (2H12), NFκB p65 (F-6), mTOR (30), AKT (B-1), and β-actin (C-2) were purchased from Santa Cruz Biotechnology (Dallas, TX, USA) and PARP1 (9542), p-IκBα (Ser32) (14D4), IκBα (9242), p-NFκB p65 (Ser536) (3033), p-mTOR (Ser2448) (5536) p-AKT (Ser473) (9271), p62 (5114), LC3B (2775) were purchased from Cell Signaling Technologies (Danvers, MA, USA). After washing with 1x TBST, the membranes were incubated with anti-mouse or anti-rabbit horseradish peroxidase-conjugated secondary antibodies for 1 h. The signals were detected using an Image Quant LAS mini (Fujifilm, Tokyo, Japan).

### 3.8. RT-qPCR 

Total RNA was extracted from HeLa cells treated with PA for 48 h with an RNeasy mini kit (Qiagen, Hilden, Germany) according to the manufacturer’s protocol. cDNA was synthesized using the iScript™ cDNA Synthesis Kit (Bio-Rad, Hercules, CA, USA) with the same amount of RNA. RNA concentration was measured using Nanodrop (Thermo Fisher Scientific, Waltham, MA, USA). Specific primers were added to the PCR reaction, including 10 μL of iTaq Universal SYBR Green Supermix (Bio-Rad, Hercules, CA, USA), 1 μL of PCR Forward Primer (10 μM), 1 μL of PCR Reverse Primer (10 μM), 1 μL of cDNA template, and 7 μL of ddH2O. The reaction was amplified through a flowing step at 95 °C for 10 min of denaturation, followed by 40 cycles of 95 °C for 15 s, and 60 °C for 60 s using a StepOnePlus Real-Time PCR system (Thermo Fisher Scientific, Waltham, MA, USA). Primer sequences (5′→3′) used in the study. BAK (F: ATGGTCACCTTACCTCTGCAA, R: TCATAGCGTCGGTTGATGTCG), BAX (F: CCCGAGAGGTCTTTTTCCGAG, R:CCAGCCCATGATGGTTCTGAT), NOXA (F: ACCAAGCCGGATTTGCGATT, R:ACTTGCACTTGTTCCTCGTGG).

### 3.9. Transfection

HeLa Cells were seeded in 12-well plates up to a density of 1 × 10^5^ cells and cultured for 24 h. RFP-LC3B plasmid was transfected into the cells using Lipofectamine 2000 according to the manufacturer’s instructions (Thermo Fisher Scientific, Waltham, MA, USA). After 12 h, the transfection medium was replaced with fresh culture medium and the cells were incubated for 24 h before PA treatment. Cells expressing RFP-LC3B were analyzed and subjected to fluorescence imaging [56,57].

### 3.10. Live-Dead Assay

The HeLa cells were seeded in 6-well plates up to a density of 1 × 10^6^ cells in each well and the cells were treated with PA (10 mM and 20 mM) for 48 h. The HeLa cell morphologies were analyzed using fluorescent dyes for both living and dead cells using the LIVE/DEAD kit (Thermo Fisher Scientific, Waltham, MA, USA). The cells were stained using EthD-1 and calcein, according to the manufacturer’s instructions. Images were taken using a fluorescence microscope (Olympus, Tokyo, Japan).

### 3.11. Cell Cycle Analysis

The HeLa cells were seeded in 6-well plates up to a density of 1 × 10^6^ cells in each well and the cells were treated with PA (10 mM and 20 mM) for 48 h. The cells were then collected and fixed in ice-cold 70% ethanol at 4 °C for 4 h. After 3 min of centrifugation at 1500× *g*, a premixed reagent containing RNAse A and the nuclear DNA intercalating dye propidium iodide (PI) was used to stain the cells, which is required to conduct analyses with the Muse Cell Cycle Assay Kit (Luminex Corporation, Austin, TX, USA). The percentage of cells in each cell cycle phase was determined with the FlowJo software (Version 10, TreeStar, Ashland, OR, USA).

### 3.12. Statistical Analysis

Statistical analysis was performed using GraphPad Prism (GraphPad Software, Inc., version 7, San Diego, CA, USA), and the values were provided as means ± SEM. The data were further analyzed through the Student’s t-test. The resulting *p*-values (* *p* < 0.05, ** *p* < 0.01, *** < 0.001) were considered statistically significant.

## 4. Conclusions

In conclusion, we demonstrated that propionate acid (PA), a SCFA, stimulates ROS accumulation in cervical cancer cells. This leads to the dysfunction of the mitochondrial membrane, which induces cell death. Our data also showed that PA blocks antiapoptotic markers and induces the proapoptotic proteins at both the gene and protein levels. In addition, PA inactivates the NF-kB pathway, which is known to regulate cell survival in cervical cancer cells by reducing p-65 and p-IkBa. Furthermore, PA inhibits mTOR/AKT and upregulates LC3B to induce autophagy. The findings of this study suggest that PA could serve as a potentially effective therapeutic option for the treatment of cervical cancer.

## Data Availability

The data presented in this study are available in the article.

## Supplementary Materials

The following are available online, Figure S1: Effect of PA on the viability of cervical cancer and normal cells, Figure S2: Paclitaxel and etoposide-induced apoptosis in HeLa cells, Figure S3: Effect of PA and 3-MA in HeLa cells.

## Sample Availability

Samples of the compounds are available from the authors.
