# Supplementary material for: Anticancer Effects of Propionic Acid Inducing Cell Death in Cervical Cancer Cells
Source: Molecules. 2021 Aug 16;26(16):4951. doi: 10.3390/molecules26164951 (PMC8399869; doi:10.3390/molecules26164951)
Supplement: Supplementary file 1 [file molecules-26-04951-s001.zip › molecules-1308079-supplementary.pdf]

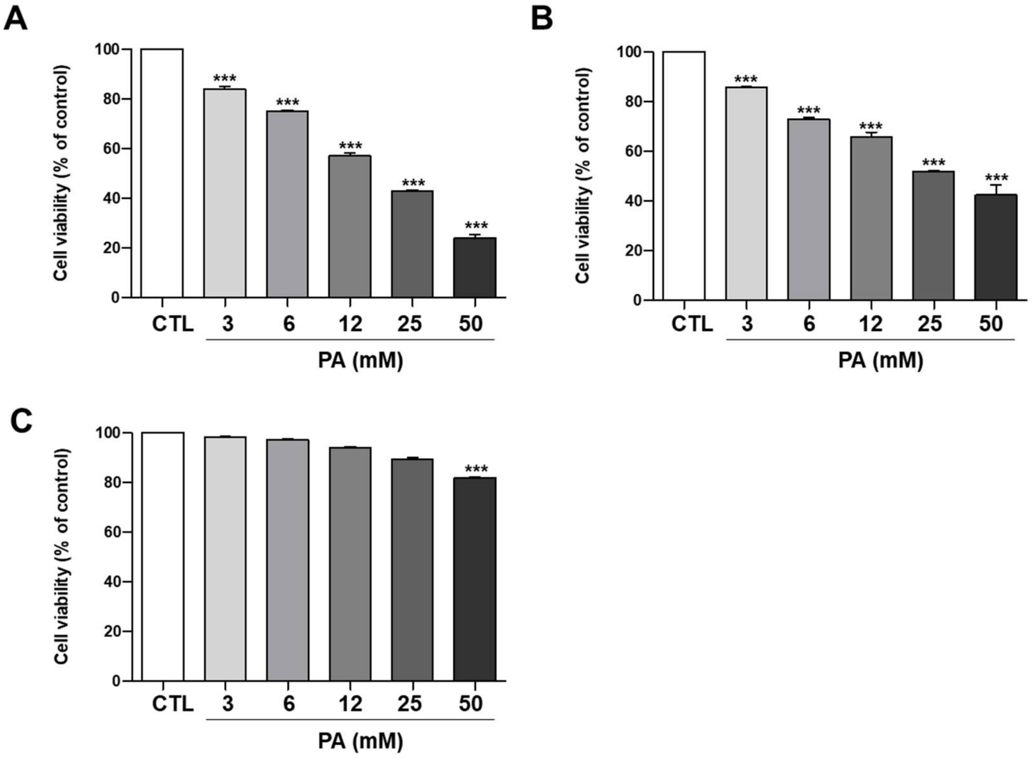

Figure S1. Effect of PA on the viability of cervical cancer and normal cells. The cervical carcinoma cell lines (A) CaSki and (B) SiHa and the normal human bronchial epithelial cell line (C) BEAS-2B were treated with various concentrations of PA. Cell viability was determined using the MTS assay 48 h after treatment with PA (n=3, *** P ≤ 0.001).

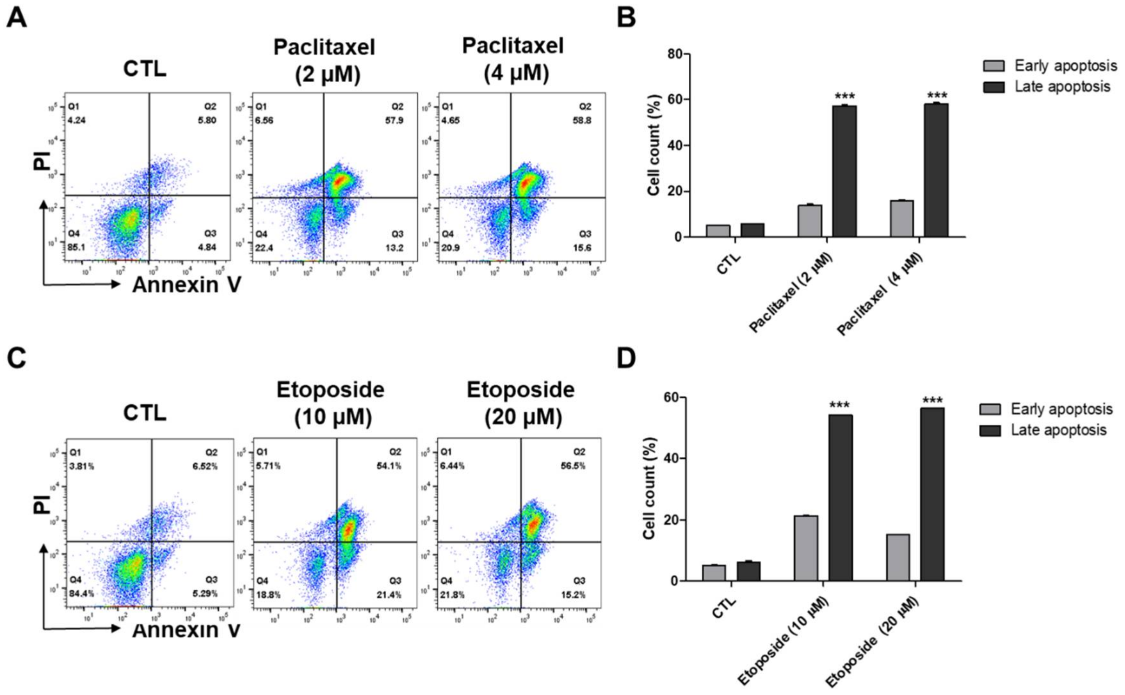

Figure S2. Paclitaxel and etoposide-induced apoptosis in HeLa cells. Apoptosis population in HeLa cells after treatment with paclitaxel (A) and etoposide (C) for 48 hours. Quantification of early and late apoptosis populations following treatment with paclitaxel (B) and etoposide (D) (n=3, *** P ≤ 0.001).

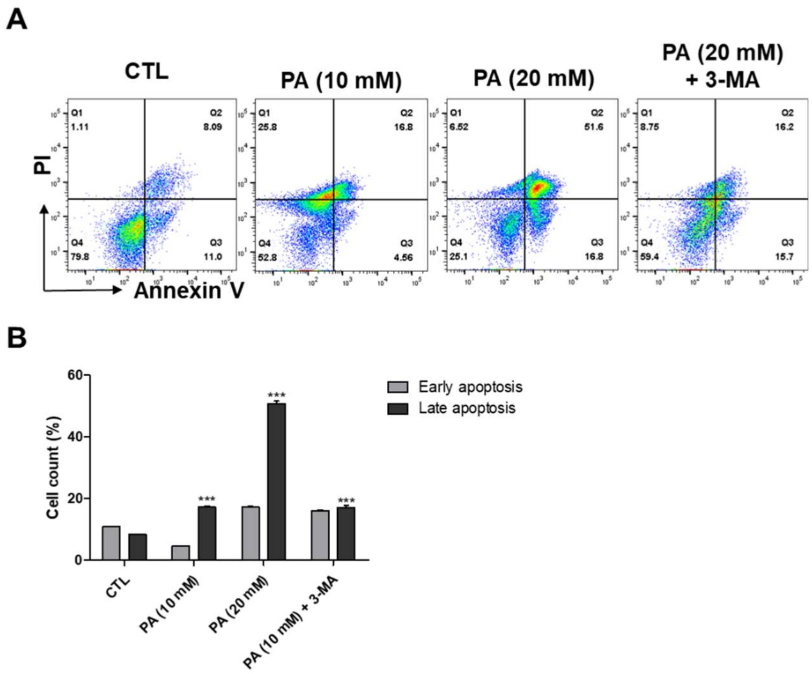

Figure S3. Effect of PA and 3-MA in HeLa cells. (A) Apoptosis population of HeLa cells. The cells were treated with 5 mM 3-Methyladenine (3-MA) for 4 hours then treated with PA for 48 hours. (B) Quantification of the early and late apoptosis populations. (n=3, *** P ≤ 0.001).
